# Chest Movements of Respiration in the Indian Female

**Published:** 1887-06

**Authors:** 


### CHEST MOVEMENTS OF RESPIRATION IN THE INDIAN FEMALE.

A report of some investigations into the subject of respiratory chest movements of Indian females, published by Dr.

Thos. J. May, in the *Therapeutic Gazette, May 16, 1887,* seem to indicate that the costal respiration in the civilized female is a purely artificial result. The examinations were made by means of a pneumograph, modified by the examiner, constructed on the model of that of Paul Bert. The subjects of the investigation were eighty-two Indian girls in the Lincoln Institution, and in each case both a costal and an abdominal tracing was taken. Thirty-three of the girls were full-blooded Indians, five one-fourth, thirty-five one-half, and two three-fourths white. Seventy-five showed a decided adominal type of breathing, three a costal type, and three in which both were about even. The author states: "In no single instance did the full-blooded Indian girl possess the costal type of breathing."

As the author suggests, the result of these investigations throws suggestive light upon what may perhaps be termed the transcendental physiological speculations of Boerhaave, Huller, Hutchinson and their many disciples.

The author further raises, and partly discusses, the question: Does this fact of the abdominal breathing among Indians, with its possible relative apex inactivity, account for the very great mortality from pulmonary tuberculosis among those tribes which have most recently come in contact with civilization? and also, what is the influence of such abdominal constriction, as is practised by our civilized female, upon the function of respiration, and upon the fact that a relatively smaller number of females die of pulmonary tuberculosis? The article from which these remarks are very freely quoted is accompanied by tracings which show in a striking manner the facts claimed, and is worthy of consideration.

